# Editorial

**Published:** 1839-10-12

**Authors:** 


### THE MEDICAL EXAMINER.

### PHILADELPHIA, OCTOBER 12, 1839.

At the late election at Philadelphia, Dr. Heintzelman was chosen Coroner. This gentleman was for some years engaged in the practice of medicine, and possesses many qualifications for the responsible office of coroner.

It is, we believe, the first time that a member of the medical profession has been elected at Philadelphia to fill an office which demands, for an adequate fulfilment of its duties, an acquaintance, at least, with the anatomical structure and medical jurisprudence. It is true that a conscientious coroner (and our late coroner was both an able and a conscientious officer) endeavours to supply the deficiency of his own knowledge by a frequent reference to a medical adviser; but this is an imperfect mode of getting at second hand that information which can be most effectually applied when possessed by the officer himself. The duties of the office are such that the coroner is often unable to consult with a physican who is familiar with the subject, and he is obliged either to dispense with medical assistance when examining a body, or to call upon a practitioner of medicine who may be quite unaccustomed to the peculiar and often difficult task of deciding a mooted point of medical jurisprudence. Yet these difficulties are of the most common occurrence,—and a neglect of duty frequently occurs, not from unwillingness on the part of the coroner to act with fidelity, but from inability to perform a task which requires a long and laborious training.

We do not think it necessary that a coroner should possess so intimate an acquaintance with medical jurisprudence as to enable him to dispense with all aid from those of his professional brethren whose studies may have been directed to a difficult department of the science; but if his original powers of mind be good, and his professional education derived from a correct source, a medical coroner is always apt to seek assistance when needed, and to aid the jury in deciding upon a multitude of cases which are familiar to a physician, but strange to those who witness them for the first time.

We believe that our views accord with those of most of our professional brethren. An office of this kind should not be given purely as a reward for political claims; it is properly unconnected with politics, and should be conferred only on those who are conversant with the questions which a coroner is almost daily called upon to decide. The subject has been recently agitated at London, and terminated in the election of Mr. Wakley, the editor of the Lancet, on the ground that as a medical man he was well fitted to discharge the duties of the office. Yet, politically speaking, his course of action was that of a prominent partisan, and at variance with the opinions of most of his constituents.

There is another matter connected with the subject, in which physicians and the public are both interested. It is this: medical men are frequently called upon to make examinations of bodies brought before the coroner, and to give their testimony as to the cause of death. We do not know whether physicians are legally obliged to perform this duty; but, at any rate, they are not generally disposed to shrink from the performance of a task which, although burthensome, seems necessary to the public good. But, for the sacrifice of time and comfort which is entailed by the exercise of that knowledge which is obtained only at a considerable expenditure of time and money, they are clearly entitled to a just remuneration; and we doubt not that a proper representation of the subject to the legislature would obtain for them a just recompense for the duties which are to be regarded as strictly professional.

